# Effects of fire disturbance on soil respiration in the non-growing season in a *Larix gmelinii* forest in the Daxing'an Mountains, China

**DOI:** 10.1371/journal.pone.0180214

**Published:** 2017-06-30

**Authors:** Tongxin Hu, Long Sun, Haiqing Hu, Futao Guo

**Affiliations:** 1 College of Forestry, Northeast Forestry University, Harbin, China; 2 College of Forestry, Fujian Agriculture and Forestry University, Fuzhou, China; RMIT University, AUSTRALIA

## Abstract

In boreal forests, fire is an important part of the ecosystem that greatly influences soil respiration, which in turn affects the carbon balance. Wildfire can have a significant effect on soil respiration and it depends on the fire severity and environmental factors (soil temperature and snow water equivalent) after fire disturbance. In this study, we quantified post-fire soil respiration during the non-growing season (from November to April) in a *Larix gmelinii* forest in Daxing'an Mountains of China. Soil respiration was measured in the snow-covered and snow-free conditions with varying degrees of natural burn severity forests. We found that soil respiration decreases as burn severity increases. The estimated annual C efflux also decreased with increased burn severity. Soil respiration during the non-growing season approximately accounted for 4%–5% of the annual C efflux in all site types. Soil temperature (at 5 cm depth) was the predominant determinant of non-growing season soil respiration change in this area. Soil temperature and snow water equivalent could explain 73%–79% of the soil respiration variability in winter snow-covering period (November to March). Mean spring freeze–thaw cycle (FTC) period (April) soil respiration contributed 63% of the non-growing season C efflux. Our finding is key for understanding and predicting the potential change in the response of boreal forest ecosystems to fire disturbance under future climate change.

## Introduction

Soil respiration (Rs) is the second-largest carbon flux in most terrestrial ecosystems—the amount of CO_2_ released by soil respiration is more than ten times that released by global fossil fuel combustion [1, 2]. It contributes 20%–40% of the CO_2_ input to the atmosphere [3]. Soil respiration is estimated to be 80–98 Pg C·yr^-1^ [4]. Therefore, slight changes in soil carbon may influence the global carbon balance. Many studies focus on forest soil respiration during the growing season [5–9], and estimated the annual soil respiration by assuming the respiration flux near zero during the non-growing season [10]. However, recent studies have shown that the average rate of forest soil respiration during the non-growing season is 0.15–0.67 μmol CO_2_·m^–2^·s^–1^ [11–16], which accounts for 2%–37% of annual soil respiration [17–20], and significantly affects the carbon balance of forest ecosystems [21–24].

Nearly 50% of terrestrial ecosystems in the northern hemisphere are covered by snow in winter [14]. Snow-covered ecosystems are vulnerable to climate change, as small variations in climate may result in large changes in snow covers [25]. Recent studies indicated that the decline in winter snow cover is coupled to the positive temperature anomalies [26, 27]. Higher soil temperatures can support more biological and chemical processes that promote soil respiration [28, 29]. In mid-and high-latitude regions (35–65°N), soil freeze–thaw cycles in spring directly affect soil physicochemical properties, organic matter decomposition, plant root systems, and microbial activities. Then, it influences the dynamics of soil respiration [30–33], and the peak value of CO_2_ release during the freeze–thaw cycles period [20, 34].

Boreal forests, the second largest forest type in the world [35], approximately occupied 14% of the global land area and function as the largest terrestrial organic carbon pool [36, 37]. The boreal forest ecosystem is sensitive to the micro or macro-scale temperature variability caused by forest structure and fire disturbance [38, 39]. Boreal forest carbon sequestration and emission is largely determined by forest fire disturbance [40–42]. Therefore, the frequent and severity of forest fires may significantly affect the carbon balance in boreal forest ecosystems [43, 44]. Carbon pools can be severely disturbed by fires [45, 46], which can significantly increases the C released to the atmosphere by forest vegetation and soil litter combustion [47]. The soil carbon loss in boreal forests after fire disturbances is not only a crucial factor of the forest carbon balance [45, 48], but also a uncertain point of the global carbon estimation [49]. Most of the uncertainty comes from the high heterogeneity and complex changes in soil environment characteristics after forest fires [50–52]. The fire duration, severity, and post-fire meteorological conditions can also have a significant influence on soil respiration after fire disturbance, and this effect can last several months to several years [53, 54]. Therefore, study of non-growing season soil respiration after fire disturbance can improve the accuracy of soil respiration estimation in boreal forest ecosystems.

The Daxing'an Mountains is the largest boreal forest distribution area in China, which mainly dominated by *Larix gmelinii* Rupr. occupied 70% of the total forests in Daxing'an Mountains [55]. The Daxing’an Mountains is also the frequent area for forest fires in China. A total of 1614 forest fires occurred in this region during the period 1965–2010, approximately average 35 times per year, according to fire records from the local government. The total burned forest area was 3.52 × 10^6^ hm^2^, mean 7.66 × 10^4^ hm^2^ per year during the period 1965–2010 [56].

In this paper, we examined the soil respiration and soil environmental factors during the non-growing season at the sites burned five year ago. The main objectives were to (1) quantify the soil respiration during the non-growing season and investigate the effect of snow covers on soil respiration in the *Larix gmelinii* forest ecosystem; (2) examine the effects of different fire severity on soil respiration during the non-growing season; and (3) explore the relationships among non-growing season soil respiration, temperature, and snow water equivalent before and after fire disturbance.

## Materials and methods

### Study site

The study area is located at the Daxing’an Mountains Nanweng River Forest Ecological Station (51°05′07″N–51°39′24″N, 125°07′55″E–125°50′05″E), China. The total study area is approximately 229,523 hm^2^ belonged to the state-owned woodland. The zonal soil is Podzol. The elevation in this area ranges from approximate 500 m to 800 m. The climate is the cold temperate continental monsoon. The average annual temperature is -3°C, with an extreme minimum temperature of -48°C. There are approximately 2500 annual sunshine hours, and the frost-free period is approximately 90 to 100 days. The annual total precipitation varies from 350 mm to 500 mm. Snow is composited of 10% to 20% of the annual total precipitation. The snowfall is mainly in December to March of the next year.

In April 2006, four forest fires were caused by lightning in Songling forest farm within the Nanweng River Forest Ecological Station. We selected three sites in each of the unburned (control), low burn severity, or high burn severity. Fire severity in the study area was classified according to the classification proposed by Keeley [57]. In the low severity area, fires burned ~25% of the understory shrubs, bark char height was 1.8–2.4 m, and 20% tree mortality occurred. The high severity area experienced the complete consumption of understory shrubs and litter and duff layers, 2.5–5.5 m bark char height, and 85% tree mortality. As the high heterogeneity of soil respiration, we established three 20 m × 20 m replicate sites within each of the burn severity areas. To accomplish all measurements of soil respiration on the same day, and thus avoid the influence of day-to-day variation, replicated sites were 200 meters within each other. The vegetation compositions in the study sites were shown in Table 1. All sites were located at the southwest aspect with the 10–15° (17–27%) of slope of and 463 m elevation. Within each severity area, nine permanent automatic data measurement systems were installed to record the soil temperature at five depth every 30 minutes during the period 2011–2012.

**Table 1:** Vegetative composition of the experimental plots.

Severity	Trees	Understory
Control (unburned)	*Larix gmelinii* Rupr.^a^, *Betula platyphylla* Suk.	*Rhododendron Simsii* Planch., *Vaccinium vitis-idaea* L., *Paris quadrifolia*, *Pyrola calliantha* H. Andr., *Vaccinium uliginosum* Linn.
Low	*Larix gmelinii* Rupr.^a^, *Betula platyphylla* Suk.	*Lespedeza bicolor* Turcz., *Rosa davurica* Pall., *Vaccinium vitis-idaea* L., *Rhododendron Simsii* Planch., *Calamagrostis angustifolia* Kom., *Maianthemum bifolium*
High	*Larix gmelinii* Rupr., *Betula platyphylla* Suk.	*Lespedeza bicolor* Turcz., *Rosa davurica* Pall., *Vaccinium vitis-idaea* L., *Rhododendron Simsii* Planch., *Calamagrostis angustifolia* Kom., *Maianthemum bifolium*

Note:

^a^ indicates that the dominant species.

### Ethics statement

The research complies with all laws of the country (China) in which it was approved by the National Natural Science Foundation of China (permit number: 31070544, 31470657). The authority responsible for a national park or other protected area of land or sea, the relevant regulatory body concerned with protection of wildlife. We state clearly that no specific permissions were required for these locations, because these locations are uncultivated land. We confirm that the field studies did not involve endangered or protected species.

### Soil respiration measurement

**Fig 1:**
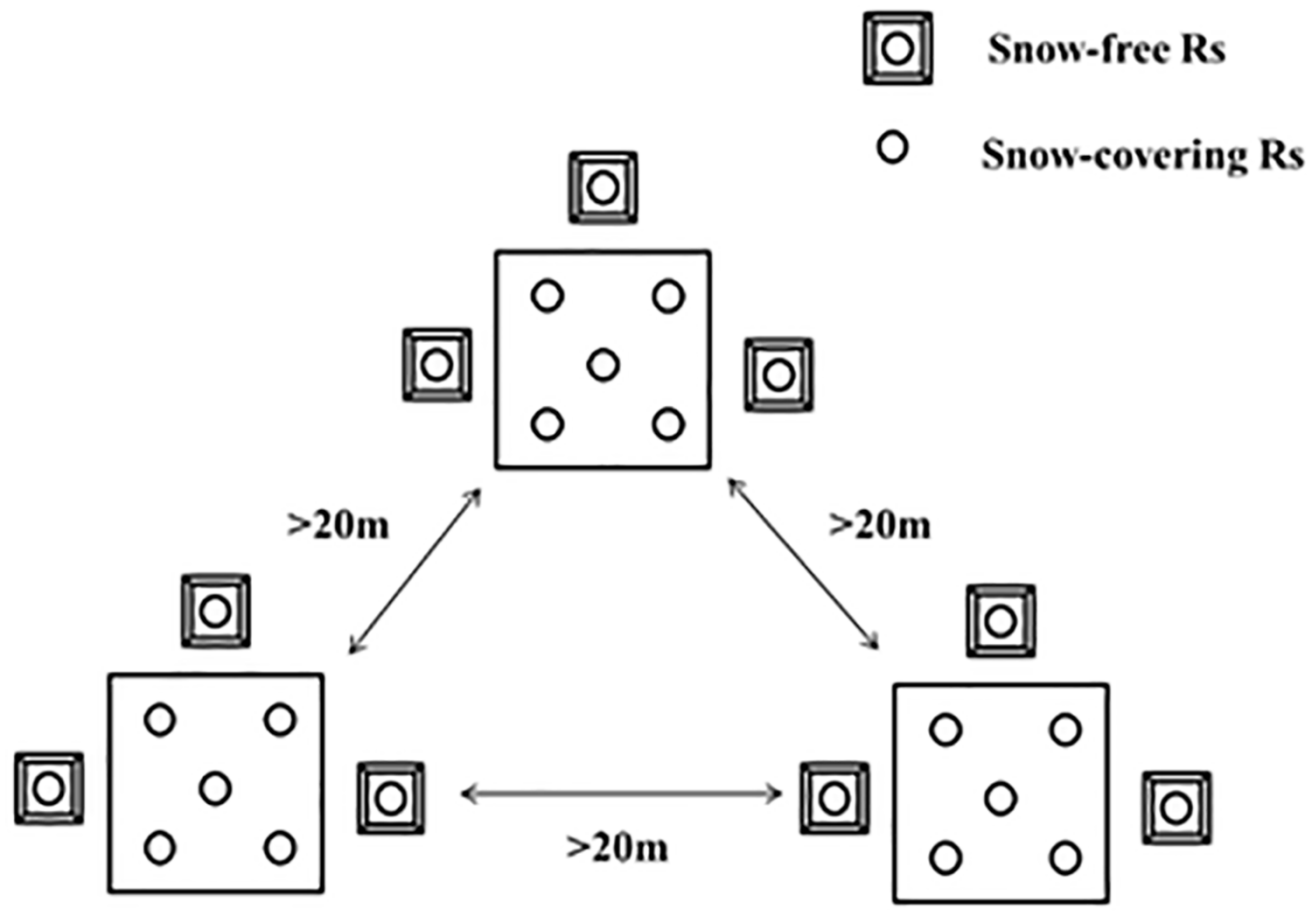
Distribution of soil respiration (Rs) rings and snow-covering soil respiration rings within a site. Each circle represents one sampling point.

**Fig 2:**
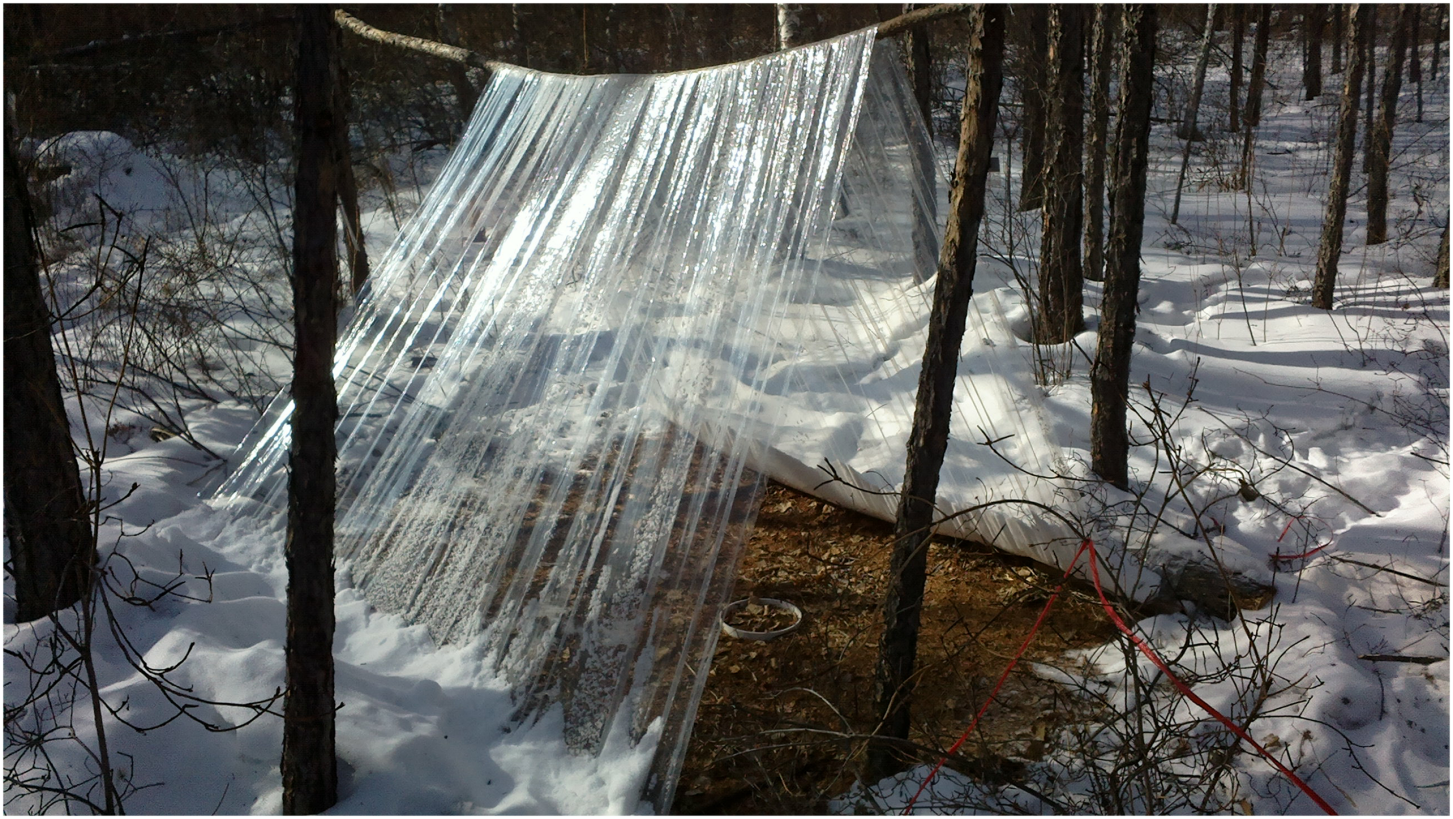
Plastic shed used for measuring soil respiration under the snow-free condition.

A portable LI-8100-1032 and LI-8100 Automatic Measuring Systems (Li-Cor Inc., Lincoln, NE, USA) were used to measure soil respiration flux. In early May 2010, five PVC (polyvinylchloride) soil rings (inner diameter 19 cm, height 7 cm) were systematically installed in each 20 m × 20 m site (Fig 1) (denoted snow-covering Rs), a total of 15 soil respiration rings in each burn severity type. The top of the PVC ring was 2–3 cm above the litter surface, and the PVC ring remained in the same position throughout the measurement period. At the beginning of November 2011, three plastic sheds (Fig 2) were randomly set up in each site to simulate a snow-free condition. Nine snow-free soil rings (3 sheds × 3 replicates; Fig 1, denoted snow-free Rs) measured soil respiration in each burn severity under snow-free conditions. The sheds consisted of a transparent plastic membrane, is open to the environment at both ends to allow light transmittance and air flow. We maintained all sheds to ensure no snow underneath them throughout the measurement period. The sheds were 1.5 m high and the horizontal size of 2 m × 2 m. A PVC soil respiration ring was placed in the middle of each shed. One end of the soil respiration ring was sharpened and pressed into the soil. The soil respiration rings were remained in the same position during the entire study period.

Eight soil respiration rings were used to measure soil respiration in each site, with five of them for snow-covering condition (Fig 1, snow-covering Rs) and three of them for snow-free sheds (Fig 1, snow-free Rs). Soil respiration was measured one time per month during the non-growing season from November 2011 to April 2012. Once snow started to thaw in early April, soil respiration rates were measured every 10 days for three measurements: early April (Apr-E), middle of April (Apr-M), and late April (Apr-L). The soil respiration measurement time was approximately 2 minutes for each soil respiration ring. Each measurement was completed between 9:00 AM and 11:00 AM. The snow-covering Rs and snow-free Rs were measured on consecutive days.

Snow thickness and mass and soil temperature (at 5 cm depth) were measured near each soil respiration ring at the same time as the Rs measurement. Snow mass was determined using a PVC cylinder with an inner diameter of 19 cm. The PVC cylinder was inserted vertically into snow until it reached soil. Then a small shovel was inserted into the bottom edge of the cylinder. The snow in the cylinder was then emptied into a plastic bag and the snow mass was measured using an electronic scale (resolution 0.01 kg, measuring range 0–45 kg). Snow thickness was the vertical depth from snow surface to soil measured with a plastic ruler [58]. Soil temperature was measured using a JM624 portable digital thermometer (resolution 0.1°C, measuring range -50 to 199°C; JinMing Instrument Co, LTD, China).

The year was divided into periods based on air temperature and soil temperature. Winter was defined as the snow-covering period, and the daily mean air temperature below 0°C lasted at least 5 consecutive days. The freeze–thaw cycle (FTC) period in spring was defined as the period from the daily maximum air temperature above 0°C (i.e., the start of snowmelt), to the daily minimum soil temperature (at 5 cm depth) above 0°C (i.e., the end of soil freezing at the 5cm depth) [20]. The growing season was defined as the period from the end of the spring FTC period to the beginning of winter. The non-growing season was defined as winter days and the FTC period, the rest days is the growing season period (Table 2).

**Table 2:** The timing, average air temperature, and average soil temperature at -5 cm of the non-growing season, spring freeze–thaw cycle (FTC) period, growing season, and annual (full year).

	Duration	Days	Air temperature (°C)	Soil temperature (°C)
Non-growing season	Nov 2011– April 2012	183	-14.94 ± 11.31	-7.53 ± 4.33
Spring FTC period	Apr-12	30	1.60 ± 8.58	-1.83 ± 2.96
Growing season	May 2011– Oct 2011	183	12.27 ± 8.6	10.74 ± 5.97
Annual	May 2011– April 2012	366	-1.26 ± 16.9	1.65 ± 10.52

### Data analysis

The data were processed and analysed using SPSS19.0 (SPSS Institute, Inc., Chicago, IL, USA). Differences in variables between burnt and control sites were tested using ANOVA and comparisons between means were performed with the least significant differences (LSD) test. All statistical analyses were performed with a significance level of 0.05.

Snow water equivalent is a measurement of the amount of water contained in snow pack. It is considered as the depth of water that would theoretically result if the whole snow pack instantaneously melted [59]. The snow water equivalent is calculated by the following equations [60]:

$$\rho_{s}=\frac{M_{s}}{V}$$ (1)

$$\text{SWE}=10\left(\rho_{s}d\right)$$ (2)

where *M*_*s*_ is snow mass (g); *V* is volume (cm^3^); *ρ*_*s*_ is the snow density (g·cm^–3^); *d* is the snow thickness (cm); and SWE is the snow water equivalent (mm). Because the snowfall mainly occurred in December of the previous year to March of the current year, and started to melt at the beginning of April. The snow water equivalent was only calculated during the main snow-covering period (Nov 2011–Mar 2012).

Model fitting for soil respiration rate and soil temperature during the non-growing season was fitted by an exponential models. The goodness-of-fit of the models were determined by the coefficient of determination (R^2^) and residual analyses. The regression model between soil respiration and soil temperature was shown as Eq 3 [61–63]:

$$\text{Rs}=\alpha \times e^{\beta \times T}$$ (3)

where Rs is the soil respiration (μmol CO_2_·m^–2^·s^–1^), T is the soil temperature at 5cm (°C), and *α* and *β* are regression coefficients.

After consideration of the common model forms [3, 64, 65], exponential models were used for analyses of fire severity, soil temperature, snow water equivalent, and the interaction effects of soil temperature and snow water equivalent during the snow-covering period (Nov 2011–Mar 2012). Logarithmic transformation of Rs was required to achieve linearity and homoscedasticity. The regression model is shown as Eqs 4 and 5:

Ln(Rs)=α+β×SWE (4)

Ln(Rs)=α+β×T+ε×SWE+ω×T×SWE, (5)

where Ln(Rs) is logarithmic transformation of Rs that was applied to achieve linearity and homoscedasticity; T is soil temperature (°C); SWE is snow water equivalent (mm), T × SWE is the interaction effect of T and SWE, and *α*, *β*, *ε*, *ω* are regression coefficients. A stepwise regression procedure was performed to remove insignificant terms (P = 0.05).

The accumulated C efflux (g C m^-2^ yr^-1^) was estimated based on parameters shown in Eq 6 [66] and calculated for both the growing and non-growing seasons.

$${\text{Accumulated C efflux}=12\times 1800\times 10}^{\text{-6}}\sum \text{Rs}$$ (6)

where 12 is the molecular weight of carbon and 1800 is a constant value (unit: second) based on the automatic data acquisition systems recording soil temperature every 30 minutes for one year.

*Q*_10_ is the temperature-sensitive coefficient representing the increase in a process as result of temperature increase at each 10°C. We used Eqs 3 and 7 to calculate *Q*_10_ [67]:

$$Q_{10}=e^{10\times \beta }$$ (7)

where *β* is the regression coefficient calculated from Eq 3 and *e* is the exponential base.

## Results

### Effects of fire disturbance on soil respiration and environmental factors

**Fig 3:**
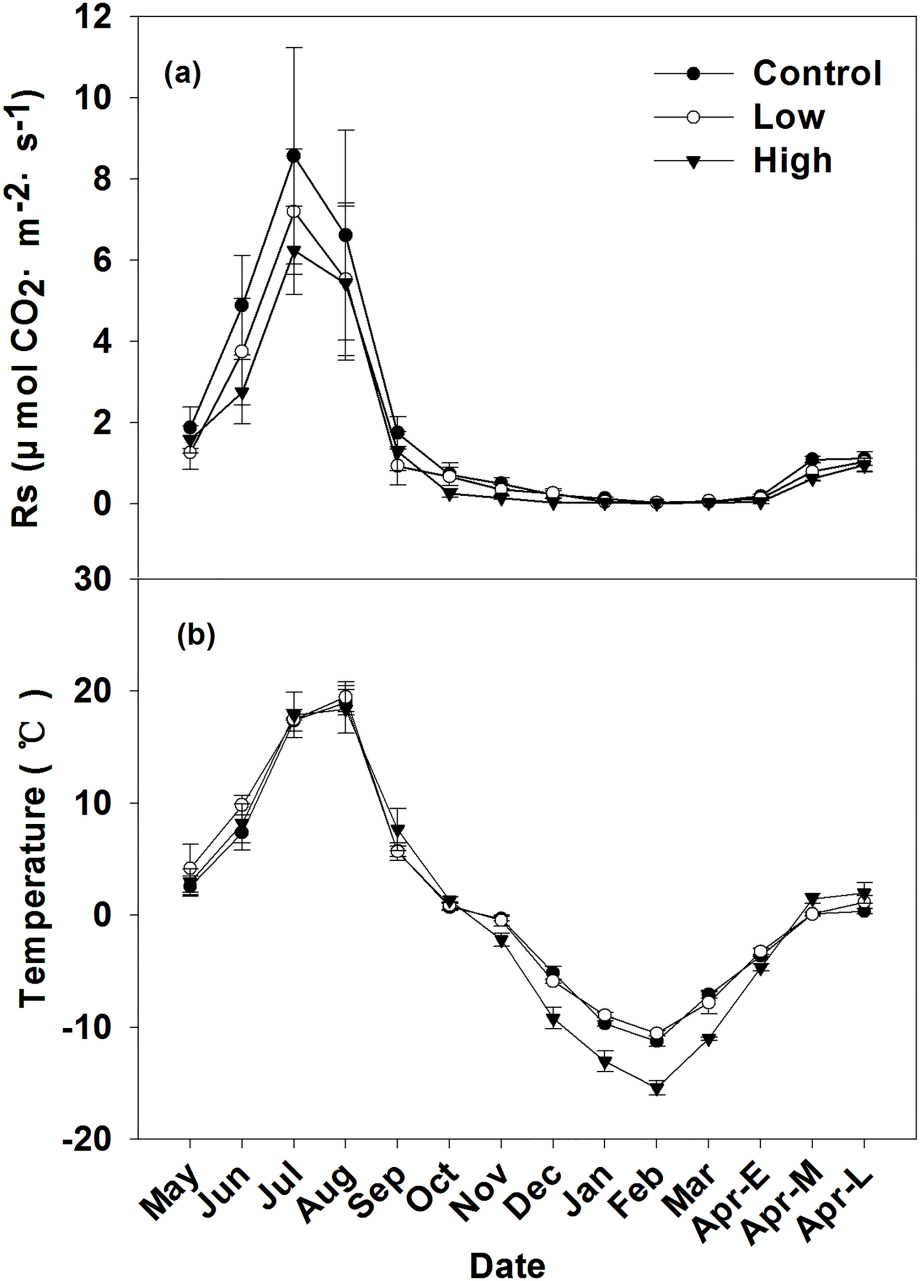
Annual (a) soil respiration (Rs), (b) soil temperature (T) of different kinds of sites (control, low burn severity, high burn severity). Mean ± se is shown in the figures.

Rs increased from May to July, then decreased until March, and increased until the end of April in all three kinds of snow-covering sites (Fig 3a). Soil temperature was relatively high in July and August and relatively low in February (Fig 3b), which was consistent with the changes in Rs. The mean Rs in the growing season were 4.06, 3.22, and 2.92 μmol CO_2_·m^–2^·s^–1^ for control, low, and high burn severity sites, respectively. The mean non-growing season Rs were 0.29, 0.23, and 0.13 μmol CO_2_·m^–2^·s^–1^ for control, low, and high burn severity sites, respectively (Table 3). The variations of soil respiration during the non-growing season (Nov to Apr-L) were similar for all kind of sites (Fig 3a). Rs in the control sites increased from 0.19 to 1.11 μmol CO_2_·m^–2^·s^–1^ from early April to late April, a six-fold increase. In the low burn severity sites, Rs increased from 0.13 to 1.03 μmol CO_2_·m^–2^·s^–1^, an eight-fold increase, from early April to late April. In the high burn severity sites, Rs increased from 0.04 to 0.95 μmol CO_2_·m^–2^·s^–1^, an increase of 24 times from early April to late April. The average non-growing season Rs at the low and high burn severity sites were 79% and 45% of that in the control sites Rs respectively. Compared with control sites, the average non-growing season Rs for high burn severity sites was significantly lower than that in the control site (P<0.05), while that of the low burn severity sites was not significantly different from control sites (P>0.05).

**Table 3:** Soil respiration(CO_2_·m^–2^·s^–1^) (Rs) and soil temperature (°C) (T) of snow-covering and snow-free conditions.

	Severity	Rs snow-covering	T snow-covering	Rs snow-free	T snow-free
Non-growing season	Control	0.29 ± 0.06	-5.76 ± 0.32	0.16 ± 0.04	-7.82 ± 0.3
Non-growing season	Low	0.23 ± 0.08	-5.71 ± 0.42	0.14 ± 0.03	-7.91 ± 0.41
Non-growing season	High	0.13 ± 0.03	-8.53 ± 0.64	0.16 ± 0.05	-9.76 ± 0.48
Spring FTC period	Control	0.19 ± 0.06	-6.7 ± 0.35	0.08 ± 0.03	-9.29 ± 0.3
Spring FTC period	Low	0.65 ± 0.17	-0.66 ± 0.34	0.43 ± 0.08	-0.11 ± 0.21
Spring FTC period	High	0.54 ± 0.08	-0.40 ± 0.55	0.54 ± 0.11	-0.03 ± 0.43

The mean non-growing season soil temperatures in the control, low, and high burn severity sites were -5.76, -5.71, and -8.53°C for control, low, and high burn severity sites, respectively (Table 3). Temperatures tended to decline from August to February, rise until July, and then stabilize (Fig 3b). The minimum soil temperature occurred in February, when the soil temperatures for control, low, and high burn severity sites were -11.24, -10.54, and -15.4°C, respectively. The maximum non-growing season soil temperatures occurred in the late April, and were 0.34, 1.16, and 1.98°C in the control, low, and high burn severity sites, respectively.

**Fig 4:**
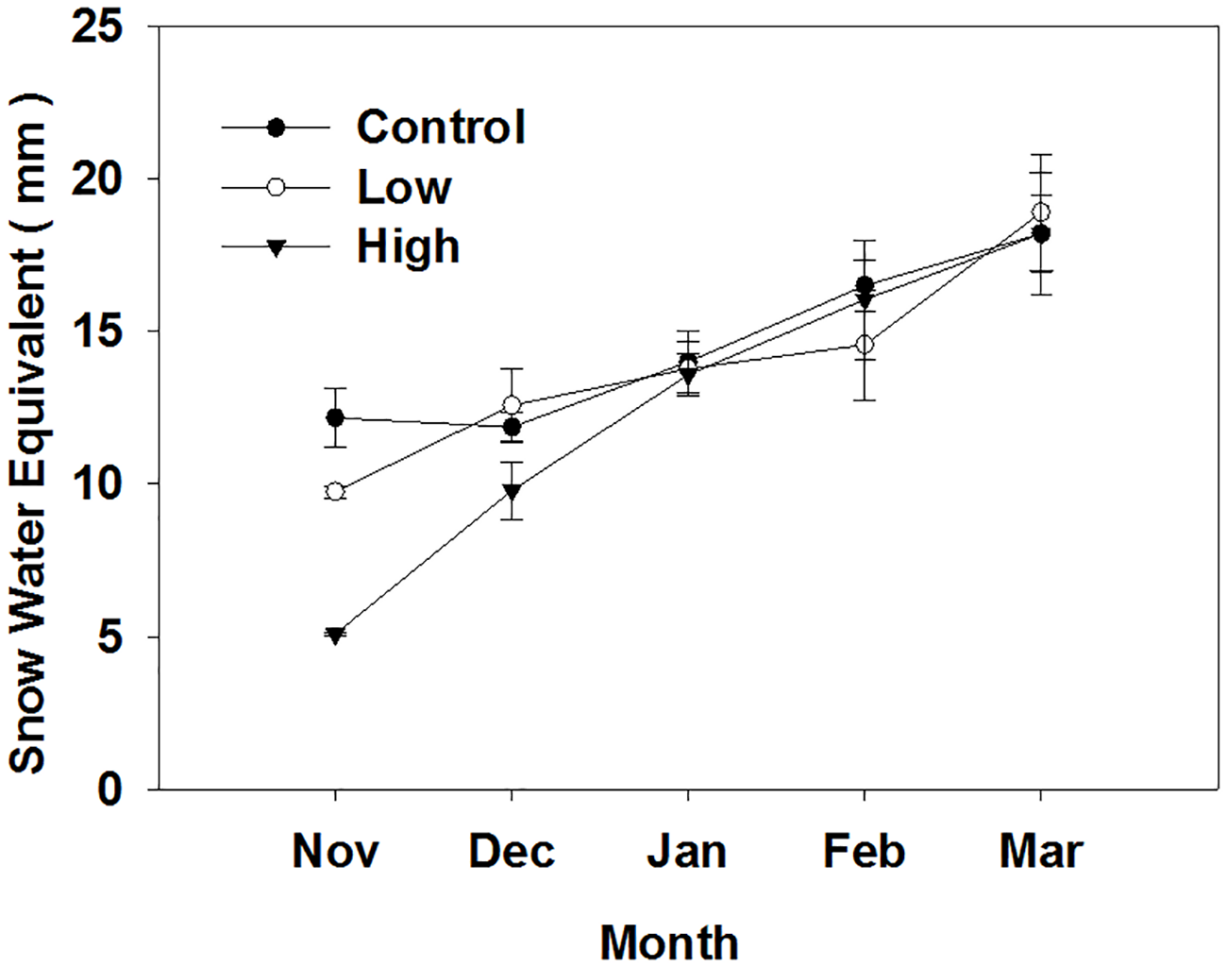
Snow water equivalent (W) of different kind of sites (control, low burn severity, high burn severity). Mean ± se is shown in the figure.

The snow water equivalent (SWE) increased with time during winter, with averages across treatments 5.1–18.9 mm (Fig 4). The SWE of control and low burn severity sites was significantly higher than that of high burn severity sites during November and December (P<0.05), but there was no measurable difference in SWE among different kinds of sites during January to March (P>0.05). The SWE accumulations of the whole non-growing season were 72.72, 69.56, and 62.68 mm for control, low, and high burn severity sites, respectively. The SWE accumulation of control and low burn severity sites were 14% and 10% higher than that in the high burn severity sites, respectively.

### Soil respiration in snow-free conditions

**Fig 5:**
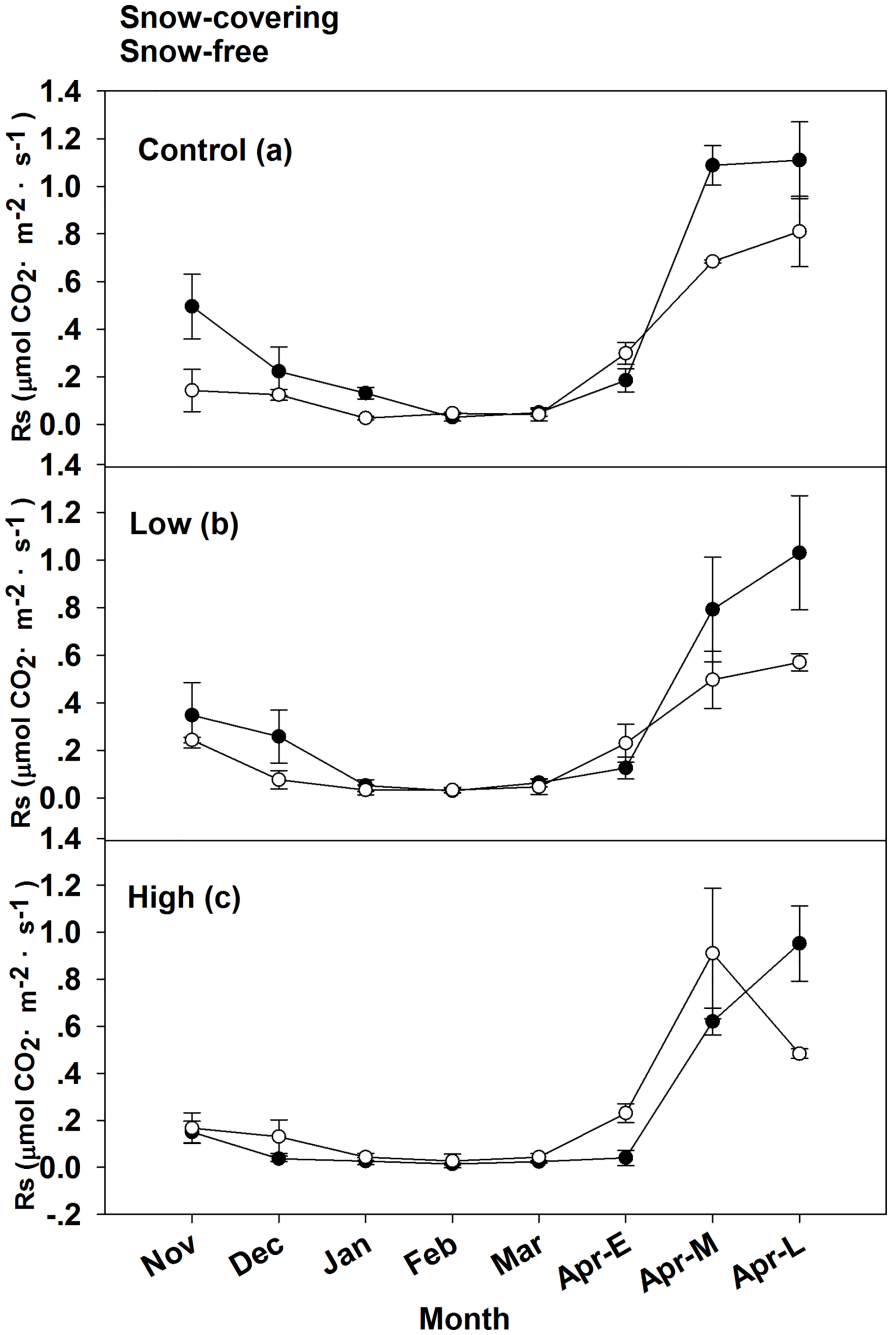
Snow-covering and snow-free soil respiration (Rs) in (a) control, (b) low burn severity, and (c) high burn severity treatments. Mean ± se is shown in the figures.

The mean non-growing season Rs for the snow-free treatment were 0.16, 0.14, and 0.16 μmol CO_2_·m^–2^·s^–1^ for control, low, and high burn severity, respectively (Table 3). The overall trends in soil respiration in the snow-free condition were similar to those in the snow-covering condition (Fig 5). Rs in different burn severity sites was significantly different (P = 0.05). The SWE significantly affected the Rs in the snow-covering and snow free treatment (P<0.05). The interactive effects of the burn severity and SWE also significantly affected Rs (P<0.05).

**Fig 6:**
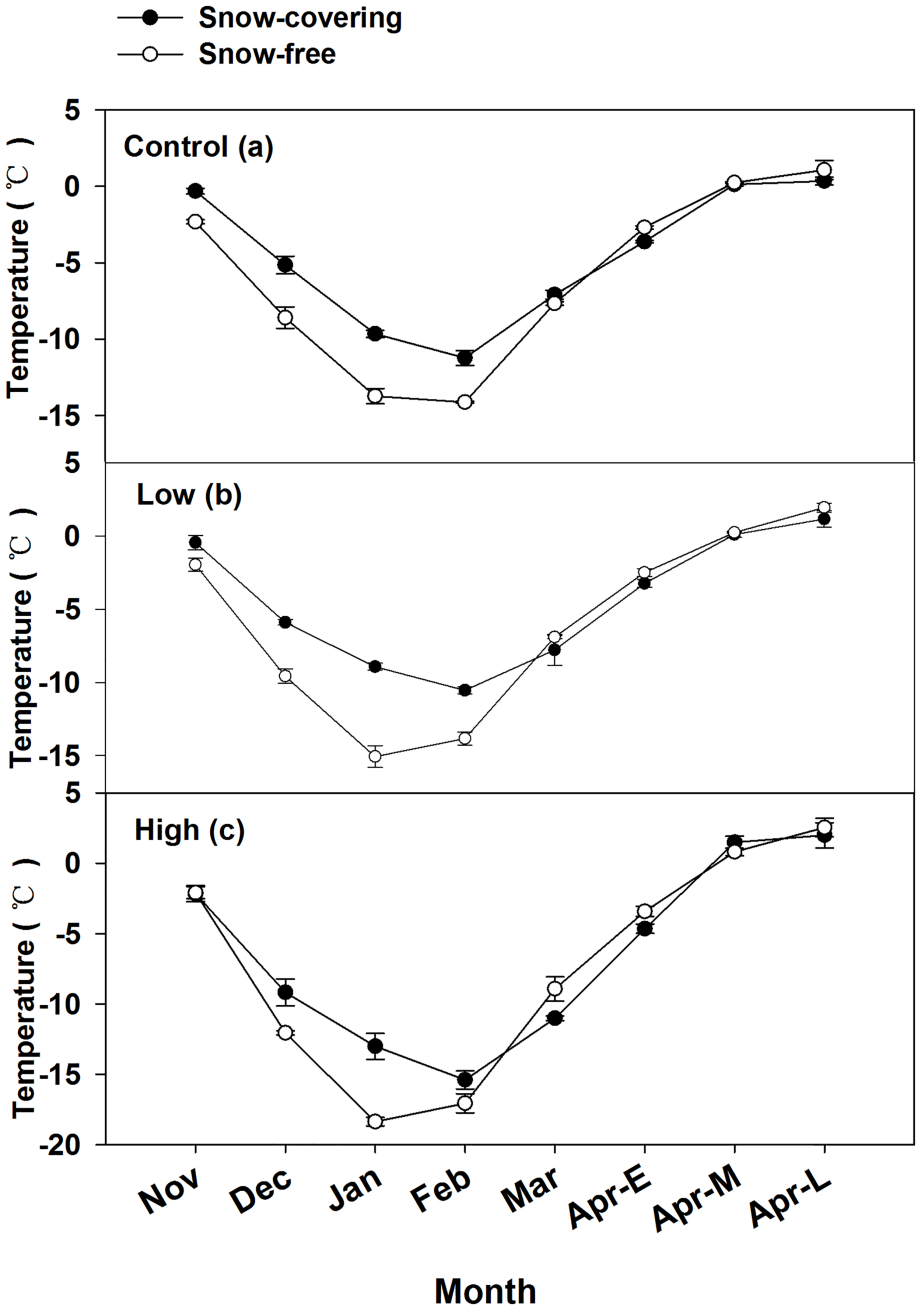
Snow-covering and snow-free soil temperature (T) in (a) control, (b) low burn severity, and (c) high burn severity. Mean ± se is shown in the figures.

The mean snow-free soil temperatures were -7.82, -7.91, and -9.76°C for control, low, and high burn severity sites, respectively (Table 3). The minimum snow-free soil temperatures all occurred in January or February, at -14.13, -15.07, and -18.37°C for control, low, and high burn severity sites, respectively. The maximum snow-free soil temperatures of all sites occurred in late April, when the soil temperatures for control, low, and high burn severity sites were 1.07, 1.93, and 2.53°C, respectively. Soil temperature in the snow-covering treatment was significantly higher than that in the snow-free treatment from November to March (P<0.05). The mean in the snow-covering Rs was twice that in the snow-free Rs during the period from November to March (Fig 6).

### Relationships between soil respiration and environmental factors

**Fig 7:**
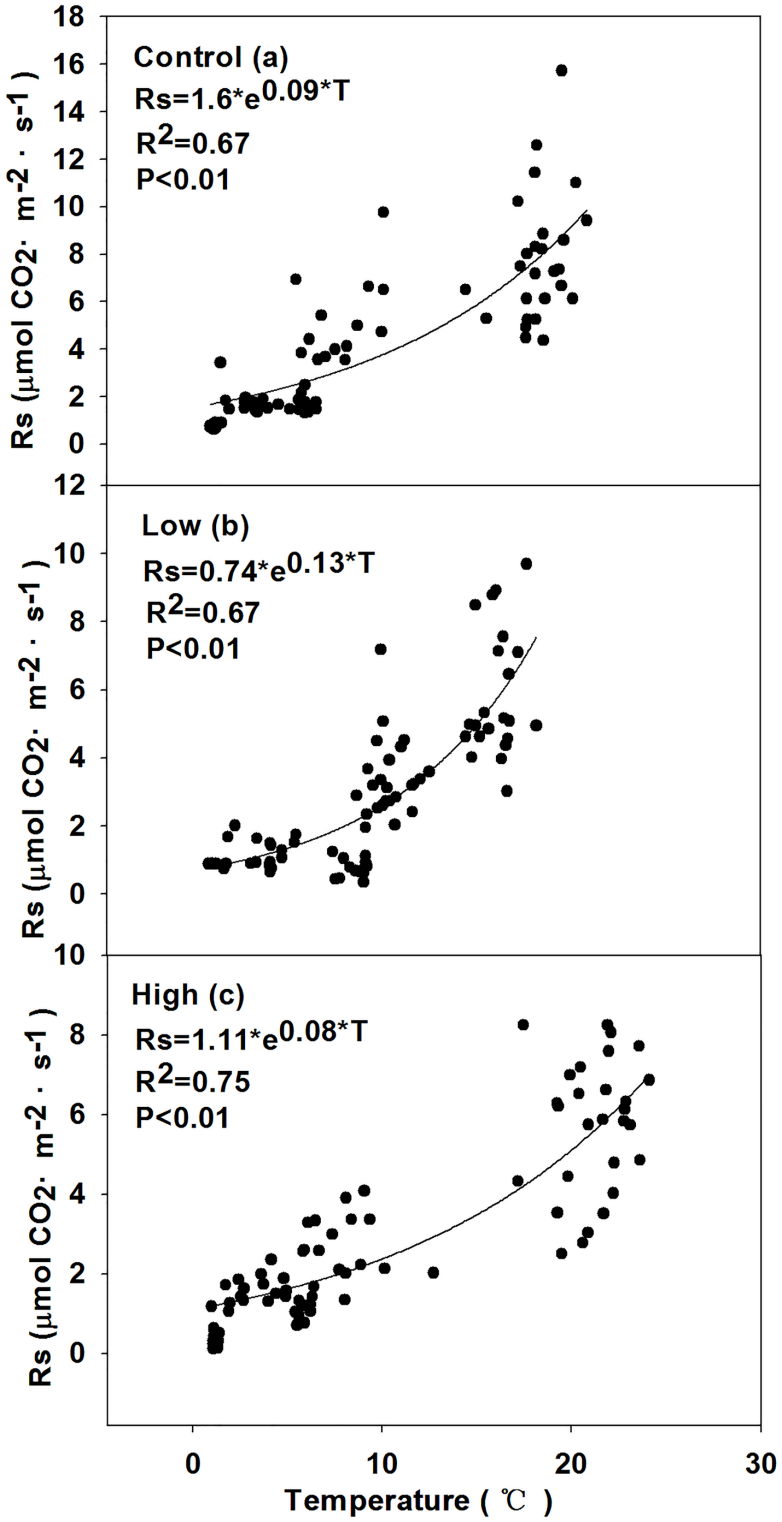
Regression of soil respiration (Rs) and soil temperature (T) fitted models of (a) control, (b) low burn severity, and (c) high burn severity in the growing season.

**Fig 8:**
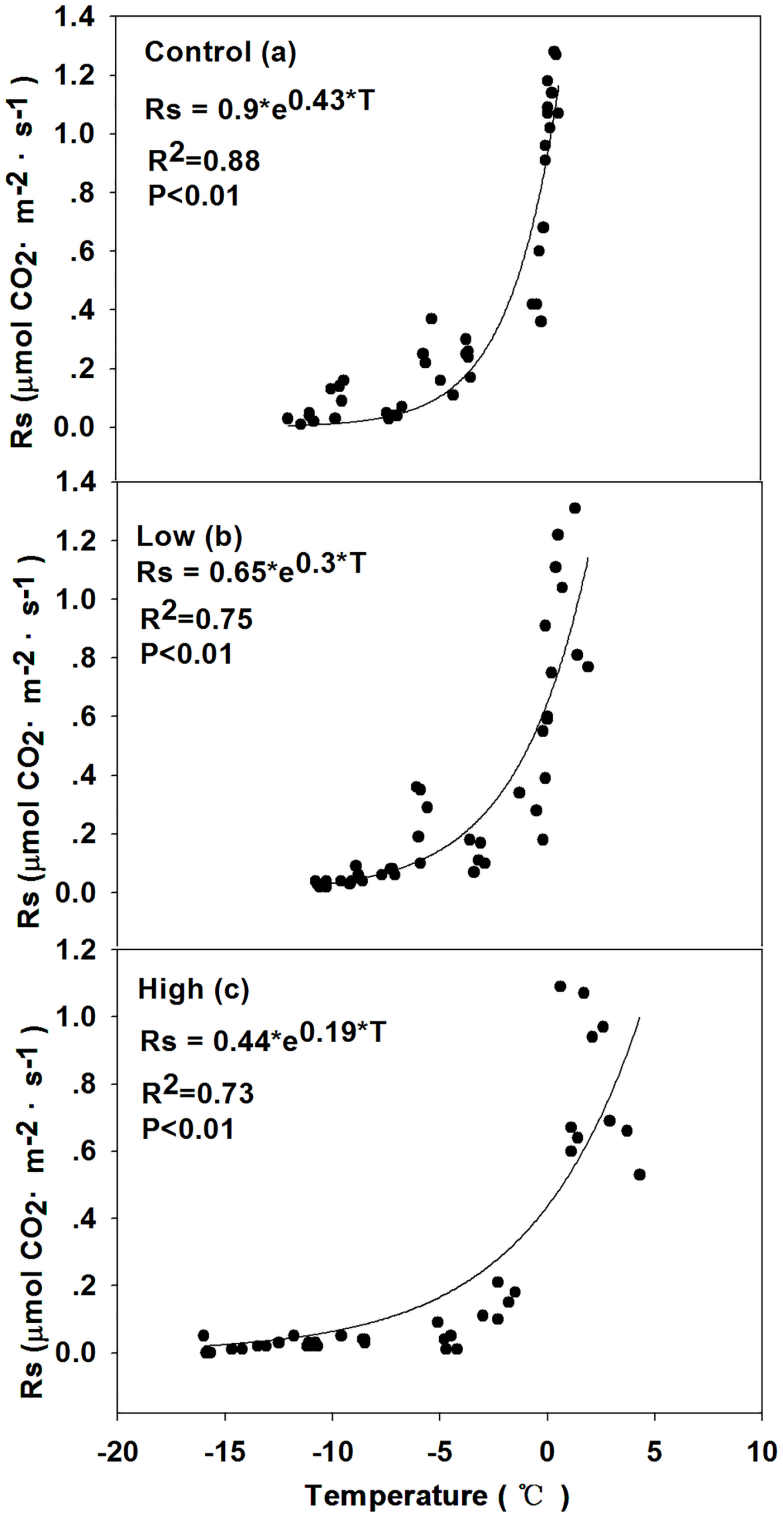
Regression of soil respiration (Rs) and soil temperature (T) fitted models of (a) control, (b) low burn severity, and (c) high burn severity in the non-growing season.

Figs 7 and 8 shown that the annual soil respiration rate significantly correlated with soil temperature in all sites. The R^2^ range of the exponential regression models during the growing and non-growing season in all sites were 0.67–0.75 and 0.73–0.88, respectively.

The exponential regressions with T as a single controlling factor of Rs were significant (P < 0.01) for all kinds of sites (control, low burn severity, and high burn severity sites), which could explain 66%–78% of the variation of the Rs in the snow-covering condition (Table 4). The exponential regressions with SWE as a single explanatory variable of Rs was also significant in all sites (P<0.01). Regression models with SWE as a single controlling factor could explain 29%–58% of the variation of the Rs in the snow-covering condition (Table 4). The models fitted with T and SWE explained 73%–79% of the variation of the Rs in different fire burn severity sites (Table 4). Temperature and snow water equivalent together improved the coefficients of regression models in control and low burn severity sites, while the exponential regressions with T as a single controlling factor could better explain the variation of the Rs in high burn severity sites (Table 4).

**Table 4:** Regression models of soil respiration(CO_2_·m^−^2·s^–1^) (Rs), soil temperature (°C) (T), and SWE is snow water equivalent (mm); T × SWE is the interaction effect of T and SWE; and *α*, *β*, *ε*, and *ω* are regression coefficients.

Severity	Model	*ɑ*	*β*	*ɛ*	*ω*	R^2^	P-value
Control	Ln(Rs) = *α*+*β*×T	-0.67	0.24			0.66	< 0.01
Low	Ln(Rs) = *α*+*β*×T	-0.73	0.25			0.74	< 0.01
High	Ln(Rs) = *α*+*β*×T	-1.63	0.22			0.78	< 0.01
Control	Ln(Rs) = *α*+*β*×SWE	2.1	-0.3			0.48	< 0.01
Low	Ln(Rs) = *α*+*β*×SWE	0.53	-0.03			0.29	< 0.01
High	Ln(Rs) = *α*+*β*×SWE	-1.56	-0.15			0.58	< 0.01
Control	Ln(Rs) = *α*+*β*×T+*ɛ*×SWE+*ω*×T×W	-0.71	†	†	0.02	0.73	< 0.01
Low	Ln(Rs) = *α*+*β*×T+*ɛ*×SWE+*ω*×T×W	-1.91	†	0.12	0.02	0.79	< 0.01
High	Ln(Rs) = *α*+*β*×T+*ɛ*×SWE+*ω*×T×W	-1.63	0.22	†	†	0.78	< 0.01

Note:

^†^ indicates that this variable of the model was not significant in an ANOVA (at the P = 0.05 level).

### Temperature-sensitive coefficient (*Q*_10_) and C efflux

The *Q*_10_ in the growing and non-growing season snow-covering and non-growing season snow-free treatments were shown in Table 5. The non-growing season *Q*_10_ in each treatment was significantly higher than that in the growing season (*P*<0.05). Burn severity in all treatment significantly affected the *Q*_10_ of the Rs (P<0.05). In the growing season, the *Q*_10_ of the Rs significantly increased and decreased after the low and high burn severity fire disturbances (P<0.05). Whether in snow-covering or snow-free condition, the non-growing season *Q*_10_ shown the similar trend that the *Q*_10_ significantly decreased with burn severity (P<0.05).

**Table 5:** The parameters and statistics of soil respiration as the exponential function of soil temperature at growing season, non-growing season snow-covering, and non-growing season snow-free stand for different burn severities. Parameter values are reported as mean ± se.

Stand type	Severity	*α*	*β*	*Q*_10_	R^2^	P-value
Growing season	Control	1.6 ± 0.23^a^	0.09 ± 0.01^a^	2.41±0.2^a^	0.67	< 0.01
Growing season	Low	0.74 ± 0.13^b^	0.13 ± 0.01^a^	3.63±0.44^b^	0.67	< 0.01
Growing season	High	1.11 ± 0.14^b^	0.08 ± 0.01^a^	2.16±0.13^c^	0.75	< 0.01
Snow-covering	Control	0.9 ± 0.04^a^	0.43 ± 0.07^a^	76.71±37.04^a^	0.88	< 0.01
Snow-covering	Low	0.65 ± 0.04^a^	0.3 ± 0.05^ab^	19.89±7.69^b^	0.75	< 0.01
Snow-covering	High	0.44 ± 0.04^b^	0.19 ± 0.03^b^	6.82±2.2^c^	0.73	< 0.01
Snow-free	Control	0.58 ± 0.03^a^	0.32 ± 0.03^a^	24.29±7.94^a^	0.93	< 0.01
Snow-free	Low	0.4 ± 0.02^a^	0.21 ± 0.02^ab^	7.77±1.05^b^	0.91	< 0.01
Snow-free	High	0.46 ± 0.06^a^	0.14 ± 0.04^b^	4.18± 1.94^c^	0.58	< 0.01

Note: P values are for overall model fit. *α* and *β* are the regression coefficients of Eq (7).

Different letters (a, b, and c) within the same column mean significant differences between different burn severity (one-way ANOVA, post hoc LSD test).

**Fig 9:**
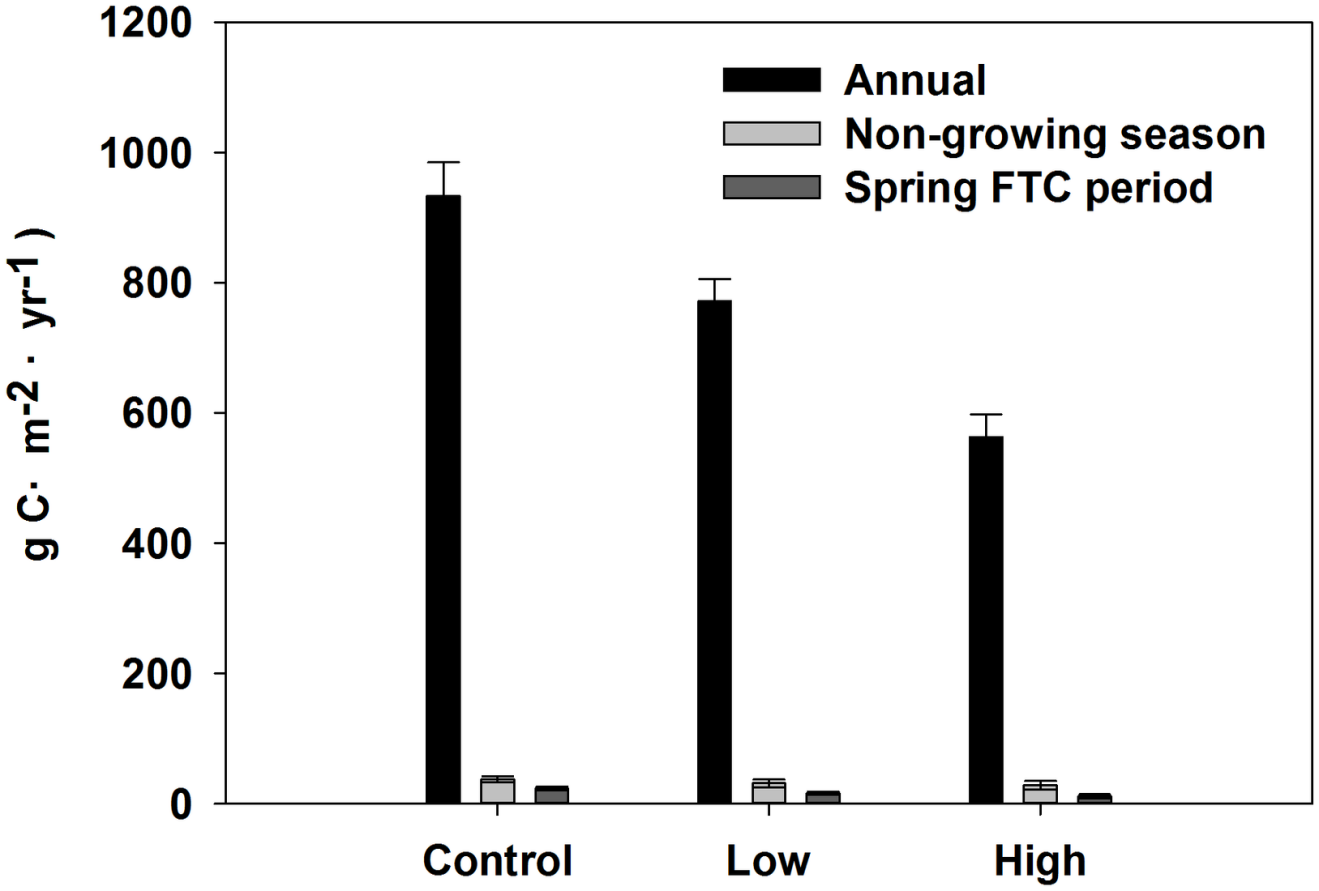
Non-growing season and annual C efflux in three different fire severity (control, low, and high).

The soil C efflux during the growing season, non-growing season, and spring FTC period (Fig 9) was calculated based on the exponential relationship between the soil respiration and soil temperature shown in Figs 7 and 8, and Table 4. We estimated that non-growing season C efflux in the control sites was 37.23 g C·m^-2^·yr^-1^, 31.3 g C·m^-2^·yr^-1^ in the low burn severity sites, and 28.24 g C·m^-2^·yr^-1^ in the high burn severity sites, contributing 4%, 4%, and 5% of annual C efflux in the control (933.11 g C·m^-2^·yr^-1^), the low burn severity (771.71 g C·m^-2^·yr^-1^), and the high burn severity (562.70 g C·m^-2^·yr^-1^) sites, respectively. Compared with control sites, the annual C efflux of the low burn severity sites was 16% lower in the non-growing season and 17% lower in the annual C efflux budget. Compared with the control sites, the annual C efflux of the high burn severity sites was 24% lower in the non-growing season and 40% lower in the annual C efflux budget (Fig 9). Meanwhile, spring FTC period C efflux was 23.41 g C·m^-2^·yr^-1^ for control sites, 16.07 g C·m^-2^·yr^-1^ for the low burn severity sites, and 11.03 g C·m^-2^·yr^-1^ for the high burn severity sites, contributing 3%, 2%, and 2% of annual C efflux of the control sites, the low burn severity sites, and the high burn severity sites, respectively.

## Discussion

### Effect of fire disturbance on soil respiration during the non-growing season

The mean non-growing season Rs in the *Larix gmelinii* forest was 0.29 ± 0.06 μmol CO_2_·m^-2^·s^-1^ (Table 3). This result was consistent with Wang et al. [20], who found that the average winter Rs rate of seven forest ecosystems in northern China was 0.28 μmol CO_2_·m^-2^·s^-1^. Many studies have found that the Rs of forest ecosystems during the non-growing season ranged from 0.15 to 0.67 μmol CO_2_·m^-2^·s^-1^ [12, 13, 16, 68]. The non-growing season Rs varies with the duration of the snow-covering period, and is also affected by climate change [69]. We should be cautious when comparing non-growing season Rs among different ecosystems because the many definitions of the non-growing season will lead to great discrepancy among different studies [70].

Fire reduces Rs to a degree dependent on the fire severity and duration [71–73]. In the non-growing season, high burn severity resulted in significantly decreased Rs, approximately 55% of that of the control sites. This result may be mainly due to the decline of autotrophic Rs in high burn severity sites. Richter et al. [74] found that Rs after fire disturbance was half that of unburnt areas in boreal forest across similar latitudes, largely because of the decline in autotrophic Rs. Severe fires have more significant impacts on autotrophic Rs than smaller fires because they cause more serious damage to plant roots. Fire restrains autotrophic Rs due to mortality of fine roots; this effect can be shrouded by the increase in heterotrophic Rs as a result of the fire in the growing season [75, 76]. Several studies have shown that heterotrophic Rs accounts for approximately 50%–70% of the total Rs during the growing season [77–79]. Heterotrophic Rs is largely caused by soil microbial activity that is partly suppressed during the non-growing season, when the majority of total Rs was provided by autotrophic Rs [24, 80, 81]. Mikan et al. [82] reported that temperature is the predominant contributor to microbial respiration in arctic tundra soil, and low temperatures will suppress the heterotrophic Rs.

The soil C efflux during the non-growing season in our forest represented approximately 4%–5% of the annual C efflux, depending on burn severity (Fig 9). Our result was consistent with the previous studies in other forest ecosystems, which accounted for 2%–37% of annual total C efflux during the non-growing season [17–20]. The annual C efflux was lower in burned treatment areas than in the control, particularly when burning was high severity. This result indicates that the influence of fire disturbances on ecosystem annual soil C efflux in boreal forests should not be ignored.

### Effect of temperature on soil respiration during non-growing season

During the growing season, the interaction between T and soil moisture content is the primary factor reported to restrict Rs [71, 83, 84]. However, the water in the soil is frozen and thus virtually ineffective for Rs during the non-growing season. Some studies have found that T is the only environmental factor that impacts Rs and have shown that Rs models using both T and soil moisture may overestimate the Rs for the non-growing season and annually [85, 86].

**Fig 10:**
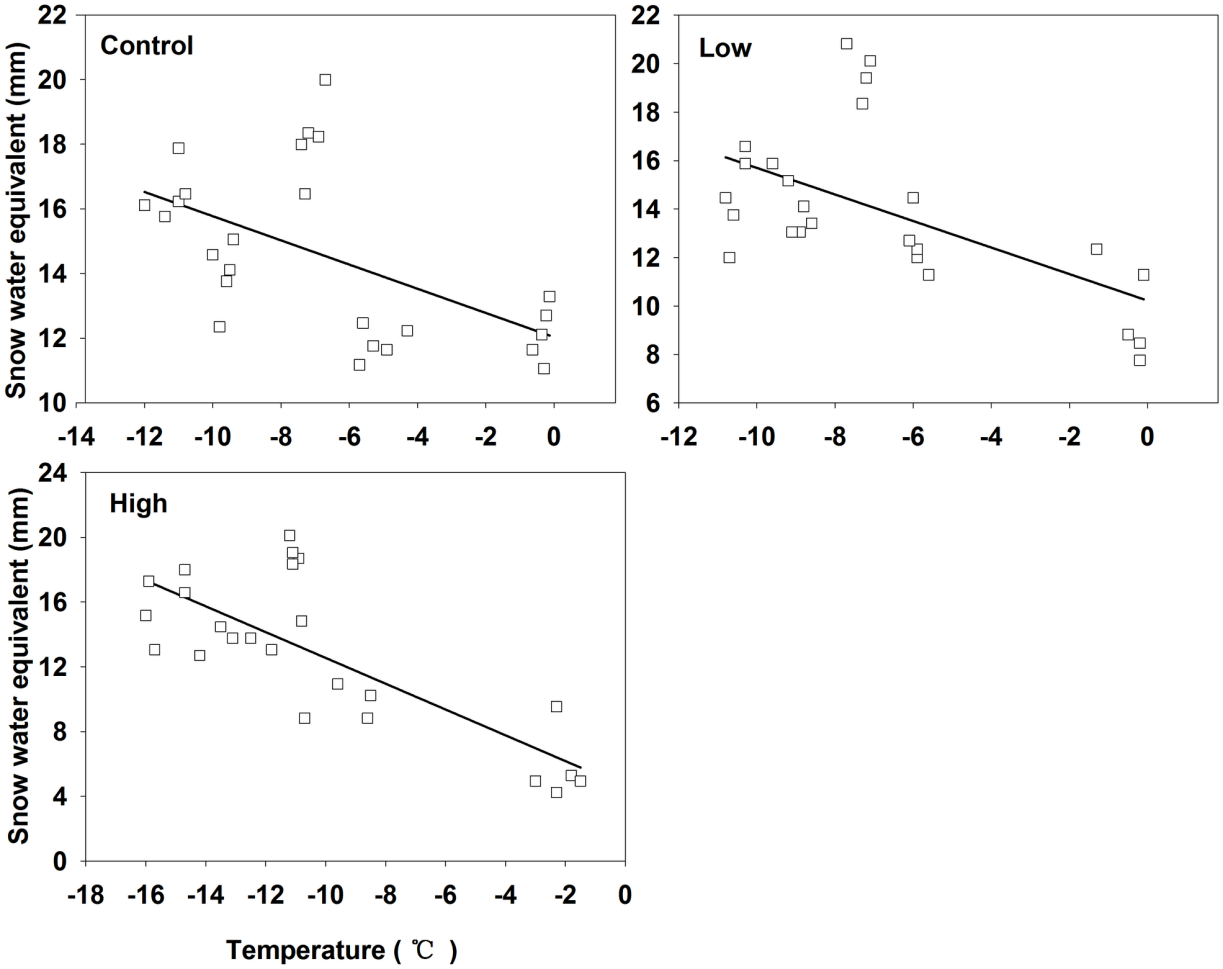
The relationship between soil temperature and snow water equivalent in three different fire severity sites (control, low, and high).

Regression models show that T is the most important abiotic factor determining Rs in the non-growing season [29, 87]. In this study, Rs in the non-growing season had a much closer relationship with T than it did in the growing season (Figs 7 and 8). Higher Rs is generally triggered by the higher temperature. Although T seems to be a better predictor than SWE, while SWE still is a significant factor affecting Rs. SWE could explain 29%–58% of the variation of Rs during the snow-covering period, which revealed the interaction of T and SWE. Fig 10 shown that with the increase of T, the SWE decreased.

**Fig 11:**
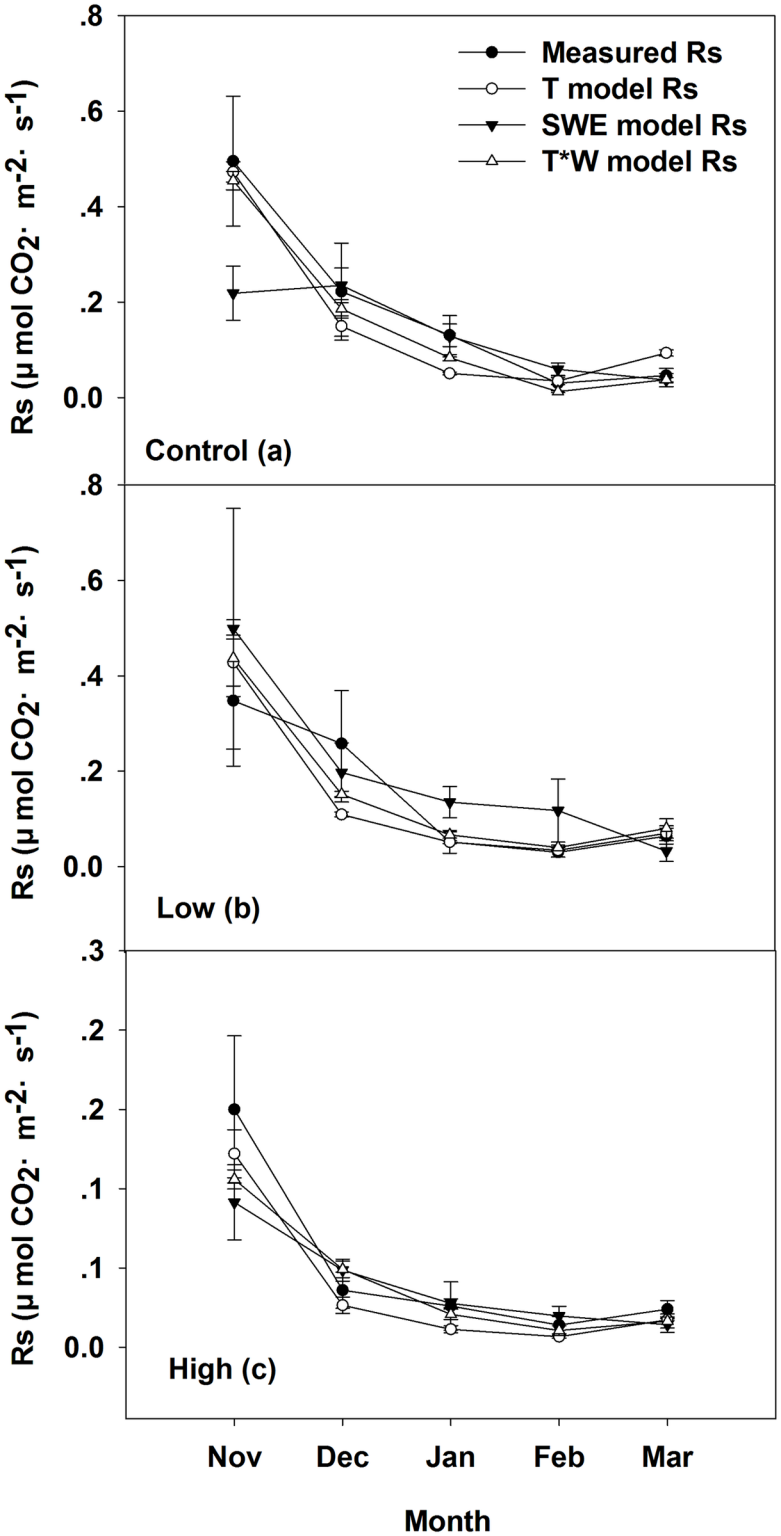
Actual measured soil respiration (●), the exponential model with temperature as a single controlling factor (○), the exponential model with snow water equivalent as a single controlling factor (▼), the model of exponential interactions of soil temperature and snow water equivalent (Δ) of (a) control, (b) low burn severity, and (c) high burn severity in snow-covering condition. Mean ± se is shown in the figures.

Fig 11 shown that the exponential model with interactions of T and SWE and the exponential model with T as a single controlling factor both could better estimate the actual measured Rs than the exponential model with SWE as a single controlling factor. Table 4 shown that the exponential model of the interaction of T and SWE were the better fit to explain the Rs in control and low severity burn sites, while the exponential model with T as a single controlling factor of Rs was the better fit to explain the Rs in high burn severity sites. There results suggested that the main controlling factor in the control and low burn severity sites was the interaction of T and SWE in snow-covering condition and the main controlling factor of high burn severity sites was still T.

The global annual *Q*_10_ of soil respiration is 2.4 [3] and many studies have indicated that the *Q*_10_ of *Larix gmelinii* forest in the northern China during the growing season ranged from 1.5 to 5.7 [88, 89]. However, the *Q*_10_ of soil respiration could be as high as 63–207 under the cold conditions [82]. Our value of the *Q*_10_ during the non-growing season was 76.71 and decreased with and the burn severity. Soil respiration during the non-growing season is more sensitive to temperature than that during the growing season (i.e. the high *Q*_10_). This may result from the high carbon availability in thawing soil from the snow-covered to snowmelt period [87, 90]. According to recent studies, the *Q*_10_ not only reflects the soil respiration sensitivity to temperature but also expresses the combined response to fluctuations in temperature, root biomass, moisture conditions, and substrate quality [83]. The variation of the *Q*_10_ after fire disturbance may result from the effects of fires on root material because the high burn severity fires will destroy root structures, cause the loss of the labile fraction of soil organic carbon (SOC) into the atmosphere, and decrease the *Q*_10_ [91, 92].

### Soil respiration change during spring FTC period

Mean Rs during the spring FTC period in this study accounted for 63% of the C efflux during the non-growing season and 3% of annual total C efflux. Wang et al. [20] performed a similar study at the Maoershan Forest Ecosystem Research Station (127°40ʹE, 45°24ʹN, 400 m a.s.l.), also in northeastern China. The much colder temperatures of our research area may be the main reason behind the lower C efflux in winter and the larger proportion of total non-growing season C efflux being produced during the spring FTC period than Wang’s findings (39%). The changes of Rs during the spring FTC period are much more complex. Snow starts to melt in early April, the Rs in the snow-free condition is higher than that in the snow-covering condition. The rapid increase in air temperature led to an increase in soil surface T and moisture [13, 93]. The increase in T greatly affects soil microbial activities, fine root growth, and the biochemical processes of soil [94]. In the early part of the FTC period (here the first 10 days of April), snow has not melt completely yet. A brief thawing results in several centimetre thick layer of ice and greatly increase the density of remaining snow. This permits greater loss of heat, and results in lower T and deeper freezing than succeeding snow cover [85].

Our findings suggest the change patterns of Rs during the spring FTC period and determine whether climate fluctuations several years after fire disturbance extend the spring FTC period and shortens the non-growing season of Daxing'an Mountains should be further explored. In addition, the mechanisms of fire disturbance on Rs and how fires change the biological (e.g., microbe, soil fauna, and plant root activity) and non-biological (e.g., soil temperature and moisture content) factors that relate to Rs in the Daxing'an Mountains also need to be studied. Our results provide a basic data for accurate understanding the effects of fire disturbance on boreal forest ecosystems under future climate change.

## Supporting information

S1 Data Soil respiration data. (XLSX)
